# Large-Scale Moth-Eye-Structured Roll Mold Fabrication Using Sputtered Glassy Carbon Layer and Transferred Moth-Eye Film Characterization

**DOI:** 10.3390/nano13101591

**Published:** 2023-05-09

**Authors:** Kazuhiro Kato, Hiroyuki Sugawara, Jun Taniguchi

**Affiliations:** 1 Geomatec Co., Ltd., Yokohama Landmark Tower, 9th Floor, 2-2-1 Minato Mirai, Nishi-ku, Yokohama 231-0022, Japan; 2 Department of Applied Electronics, Tokyo University of Science, 6-3-1 Niijyuku, Katsushika, Tokyo 162-8601, Japan

**Keywords:** moth-eye structure, nanoimprint lithography, roll-to-roll, glassy carbon, oxygen plasma, porous alumina, thin film, antireflection

## Abstract

Currently, there is high demand for the development of a highly mass-producible technology for manufacturing moth-eye-structured films with an antireflection function. Conventional moth-eye-structured films have been produced by roll-to-roll (RTR) ultraviolet nanoimprint lithography (UV-NIL) using porous alumina, but the process of manufacturing the roll mold with aluminum is both complicated and time-consuming. To solve this problem, we proposed a sputtering process for forming a thin film of glassy carbon on a roll substrate and fabricated a moth-eye structure through the irradiation of oxygen plasma. A glassy carbon (GC) moth-eye-structure roll mold with a uniform reflectance of less than 0.1% over a length of 1560 mm was fabricated following this method. In addition, a superhydrophobic moth-eye-structured film was produced by RTR UV-NIL using the proposed roll mold, which exhibited a reflectance of 0.1%. In this study, a moth-eye-structure roll using porous alumina was compared with a film transferred from it. The GC moth-eye-structure roll mold was found to be superior in terms of antireflection, water repellency, and productivity. When the proposed large-area GC moth-eye-structured film was applied to window glass, significant anti-reflection and water-repellent functionalities were obtained.

## 1. Introduction

Display devices such as liquid crystal displays and digital signage are widely used in homes and public and commercial facilities, and these displays are becoming larger. A key problem related to these devices is that visibility deteriorates because of the reflection of external light. To solve this problem, it is necessary to attach a film with an antireflection function to a display surface. Methods for preventing light reflection include applying a multilayer coating [1,2,3] and forming an antireflection structure. The former approach has been used in a wide range of fields, leading to the development of mass-production technology. However, multilayer coatings present the disadvantage that reflectance increases at a specific wavelength and depends on the angle of the incident light. Antireflection structures can be employed to overcome this drawback. One such structure involves a biological imitation of the pattern that occurs naturally on moth eyes [4], referred to as the moth-eye structure. Its shape comprises conical structures with diameters below the wavelength of light, which are densely arranged. In the visible light region of the spectrum, it is possible to prevent reflection (reflectance of approximately 0.1%) with a structure diameter of less than 140 nm and reflection at a wide incident angle [5,6]. Two methods are available for producing this moth-eye structure: the anodization of alumina [7,8,9,10] and irradiation of glassy carbon (GC) with oxygen ions [11,12,13,14].

Anodized alumina (porous alumina) is produced by immersing aluminum in a chemical solution and applying an electric field to enable processing in a wet environment (wet type). In contrast, GC is a method of irradiating an oxygen ion beam in a vacuum and is performed in a dry environment (dry type). In addition, it is necessary to fabricate a moth-eye structure on a roll plate with a width of 1560 mm and a diameter of 300 mm to accommodate an increasing number of display devices. A previous study describes the successfully increased size and mass production of anodized alumina [15]. However, in this process, aluminum is first anodized, etched, and then anodized and etched again. This is a complicated process with limited reproducibility. Therefore, the cost of producing a roll plate using this approach is correspondingly high.

To solve this problem, this study focused on the GC method. In this approach, the surface becomes rough, and a moth-eye structure is formed simply by irradiating the GC with an oxygen ion beam. However, because GC is a carbon-based material that is fired in a process similar to that of powder metallurgy, it is difficult to increase the area of the GC substrate and produce a large rolling shape. Therefore, we developed a method to form a moth-eye structure by sputtering a GC thin film onto a substrate, followed by irradiation with an oxygen ion beam [16]. In this study, the proposed method was employed using a large-scale roll mold with a moth-eye structure, and the resulting transfer film was evaluated by roll-to-roll (RTR) ultraviolet nanoimprint lithography (UV-NIL). NIL is suitable for obtaining nanoscale patterns [17,18,19,20,21,22,23,24,25,26,27,28,29,30,31,32,33,34,35,36,37], and RTR-UV-NIL can produce high-throughput nanoscale patterns [38,39,40,41,42,43,44,45,46,47,48,49,50]. In addition, anodized alumina roll molds and GC roll molds were prepared to compare their producibility and anti-reflection properties.

## 2. Materials and Methods

### 2.1. Fabricating a Moth-Eye-Structured Roll Mold Using Anodization

The original roll was a 6000 series aluminum roll with a diameter of 300 mm and a length of 1560 mm. Because the surface of the original aluminum plate had poor homogeneity, tantalum pentoxide (Ta_2_O_5_) was deposited at 200 nm as an adhesion layer using the sputter film formation method. Aluminum (containing 1 wt% titanium) was then deposited at 1000 nm by the sputter film formation method. The formed roll was immersed in a chemical solution in a large self-made water tank, and anodization and wet etching were repeated to form a moth-eye structure. Because the moth-eye structure has a tapered shape, only vertical holes were formed during the anodization process. Therefore, after a vertical hole was formed in the depth direction by anodization, wet etching was performed as a diameter expansion process. After further anodization, a vertical hole smaller than the hole diameter was formed from the bottom of the hole, which had a diameter that expanded, forming a stepped and tapered shape. Furthermore, a smooth tapered shape was obtained by repeated wet etching and anodization. These processes are detailed in a previous report [8].

The anodization step was conducted by connecting an aluminum roll mold to the anode in a 0.3% aqueous solution of oxalic acid (H_2_C_2_O_4_) at a temperature of 10 ± 1 °C and applying a voltage of 80 V for 120 s. A regulated power supply (Kikusui Electronics Co., Ltd., Yokohama, Japan, PAN110-5A) was used as the power source. Wet etching was performed by immersion in 1 mol/L of phosphoric acid (H_3_PO_4_) at a temperature of 30 ± 1 °C for 900 s. Anodization and wet etching were performed in separate water tanks. To obtain the moth-eye structure, the anodization and wet-etching processes were repeated five times under the above conditions, and finally, the anodization process was performed.

### 2.2. Fabricating a Moth-Eye-Structured Roll Mold Using a GC Thin Film

**Figure 1:**
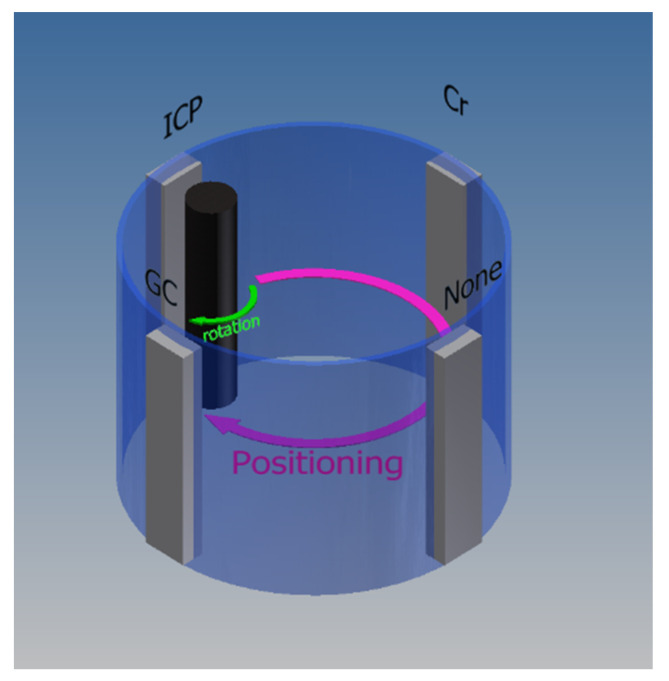
Schematic illustration showing the proposed sequential GC moth-eye roll mold fabrication machine.

A 6000 series aluminum roll with a diameter of 300 mm and length of 1560 mm was used for the original roll to form the GC thin film, which was the same as that described in Section 2.1. Because both film formation and the processing of GC thin films are dry processes, we developed a device able to continuously perform these processes, as shown in Figure 1.

As shown in Figure 1, the roll mold could form a thin film, which was irradiated with an oxygen ion beam while rotating in the circumferential direction at 2.5 rpm.

Because the adhesion between the aluminum roll and GC was poor, chromium (Cr) was employed as an adhesion layer before the GC thin film was formed. A sputter film formation method with the direct current discharge was used to form Cr and GC layers. The Cr and GC targets were plate-shaped, and the Cr target (purity 99.9%, Tosoh) was 127 mm wide, 5 mm thick, and 1575 mm long. The GC target (Nisshinbo Chemical Co., Ltd., Tokyo, Japan) with a length of 1578 mm was obtained by arranging six tiles with widths of 127 mm, thicknesses of 5 mm, and lengths of 263 mm. The distance between the target and roll was 70 mm. A Pinnacle Plus (Advanced Energy, Denver, CO, USA) source was used as the power source for DC discharge, and the target was connected to the cathode, which was connected to the anode to form a sputtered film by DC discharge. The conditions for sputtered film formation were as follows: an ultimate vacuum degree of 1 × 10^−3^ Pa for both Cr and GC, an argon flow rate of 2000 sccm for the discharge gas, a vacuum degree of 5 × 10^−1^ Pa for film formation, and no heating of the roll plate. Sputter film formation was performed using a DC power supply with an input power of 3.5 W/cm^2^ per unit area during Cr film formation. During GC film formation, DC power was applied in the pulse application mode, and sputtering film formation was performed at 7.5 W/cm^2^ per unit area.

The film thickness was measured using a stylus-type film thickness meter (Bruker AXS, Dektak XT, Billerica, MA, USA); Cr was filmed at 500 nm, and GC was filmed at 2000 nm.

Reactive-ion etching (RIE) using inductively coupled plasma (ICP) was used to form the moth-eye structure of the GC thin films. ICP may be used to obtain high-density plasma by a simple method using a coil. However, when processing a large substrate, it is necessary to use a large antenna (coil), and the antenna power will become high. Because the roll plate used in this study was as long as 1560 mm, an antenna covered with a dielectric was placed in a vacuum chamber, and a high-frequency current was passed to generate high-density plasma, which resulted in low inductance. Processing was performed using a multi-antenna ICP-RIE, in which eight antennas (low-inductance antenna, LIA, manufactured by EMD Co., Ltd., Darmstadt, Germany) were arranged.

The dimensions of one LIA antenna were 110 mm in width and 6.35 mm in length, and the dielectric covering the antenna in the vacuum chamber was 170 mm in width and 80 mm in length. Eight antennas were arranged vertically in a straight line at equal intervals from the upper end of the roller. To drive the antenna, a 13.56 MHz high-frequency (radio frequency, RF) power supply (SP-3RKI, Kyosan Electric Manufacturing Co., Ltd., Taichung, Taiwan) and blocking capacitors were used. As one RF power supply could drive two LIA antennas, four RF power supplies were employed. For the roll version, 200 W of DC power was applied at 100 kHz in the pulse application mode using Pinnacle Plus (Advanced Energy). The sample was rotated at 2.5 rpm as in the case of film formation so that the entire side surface of the roll was processed. The distance between the ICP-RIE LIA antenna and roll plate was 70 mm. The processing conditions were as follows: ultimate vacuum degree of 1 × 10^−3^ Pa; oxygen flow rate of 1450 sccm, which was used as a reactive gas to the GC; vacuum degree of 6 × 10^−1^ Pa at the time of film formation; and no heating of the roll mold. The power input of the RF power supply during processing was 0.78 kW per unit.

### 2.3. Moth-Eye-Structured Film Transfer Method Using RTR UV-NIL

RT-800U2PL equipment (Toshiba Machine Co., Ltd., Tokyo, Japan) was used for the RTR UV-NIL. The RTR UV-NIL sent out the film from the roll, coated the film with an ultraviolet curable resin, irradiated it with ultraviolet light while pressing it against the roll mold, transferred the inverted shape of the roll mold to the resin, and placed it on the film. This method involves winding the resin pattern transferred to the surface with another roll and is suitable for large-area applications and mass production because it enables continuous and seamless transfer. In this study, a cellulose triacetate (TAC) film (Fuji Film Co., Ltd., Tokyo, Japan) was used as the substrate for transfer. The thickness of the TAC film was 50−188 μm, the width was 700−1490 mm, and the film was rolled into a rolled form. The experimental conditions at the time of transfer were a transport speed of 5.0 m/min, a UV exposure of 800−1000 mJ/cm^2^, and UV TP TAC Clear OR006-5 UV-curable paint (Origin Electric Co., Ltd., Oyama City, Japan) as a UV-curable resin. Because the roll mold had a moth-eye structure with nanoscale patterns, adhesion could occur during transfer to the resin. Mold-release processing was essential to prevent this adhesion. The mold-release process used in this study was performed as follows: Optool UD-509 (Daikin Industries, Ltd., Osaka, Japan), a fluorine-based mold-release agent, was immersed in a solution diluted 400-fold with Galden (diluting solvent, manufactured by Solvay, Brussels, Belgium) for 10 min, which was then removed, air-dried, and baked at 150 °C for 1 h. The temperature was lowered to room temperature through natural cooling, and the residue was then removed by rinsing with a Galden. Both the porous alumina roll mold and GC thin-film roll mold were released using this method.

### 2.4. Roll Mold and Transfer Pattern Evaluation

Scanning electron microscopy (SEM) equipment, JSM-6700F (JEOL Ltd., Akishima, Japan), was used to confirm the shape of the roll mold and transfer patterns. The reflectance and haze of the roll plate and film on which the moth-eye structure was formed were measured using a HGM-2DP device (Suga Test Instruments Co., Ltd., Tokyo, Japan). The contact angle between water and hexadecane on the moth-eye-structured film was measured using a CA-X contact angle meter (Kyowa Surface Chemistry, Niiza, Japan). Elemental analysis of the moth-eye films was performed using energy dispersive X-ray spectroscopy (EDS). The EDS equipment used was an ULTIM MAX100 device with the AZtecLive v6.0 software (Oxford Instruments Co., Ltd., Abingdon, UK).

## 3. Results

### 3.1. Roll Mold Shape and Reflectance Characteristics

**Figure 2:**
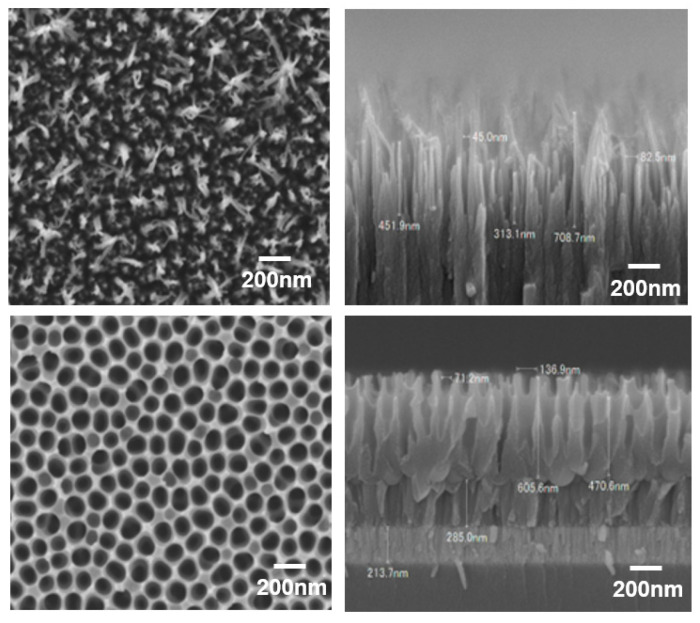
Fine structure of the processed roll plate surface. SEM images of the GC roll mold: top view (**upper left**) and cross-section (**upper right**). SEM images of the Al roll mold: top view (**lower left**) and cross-section (**lower right**).

An SEM image of the microstructure processed into a GC roll mold by the dry process is shown in Figure 2. An SEM image of the microstructure that was processed into an aluminum (Al) roll mold using the wet process is also shown in Figure 2.

As shown in Figure 2, the GC roll mold had a needle-like structure, and the Al roll mold had a conical recess shape. The Al roll mold underwent anodization and wet etching five times, and in this process, it formed a tapered depression. As shown in Figure 2, the diameter, pitch (distance between the deepest parts of the conical dent), and depth of the conical dents were measured and averaged at 10 points. Conical dents with a diameter of 119.1 ± 2.9 nm, a pitch of 139.8 ± 3.1 nm, and a depth of 413.1 ± 7.9 nm were formed. In the case of the Al roll mold, the roll mold surface exhibited an inverse pattern of the moth-eye structure, indicating high reflection. When conical dent patterns are transferred by UV-NIL, they become conical protrusions, and this shape becomes a moth-eye structure with a low-reflection surface.

However, in the case of the GC roll mold, because it had a needle-like structure, the transfer shape after the UV-NIL treatment also presented needle-like structures. As shown in Figure 2, the diameter, pitch (distance between the centers of the needles), and height of the needle-shaped structures were measured and averaged at 10 points. Needle-shaped structures with a diameter of 56.4 ± 1.8 nm, a pitch of 125.9 ± 2.3 nm, and a height of 361.8 ± 16 nm were formed. Compared to the Al roll mold surface, the diameter and pitch were low. When a carbon-based material is irradiated with an oxygen ion beam, its surface becomes rough after processing, owing to its high reactivity. This surface is often porous. However, because an oxygen ion beam was used in this study, sharpening could have occurred because of the angular dependence of the ion beam splutter rate, resulting in a needle shape.

**Figure 3:**
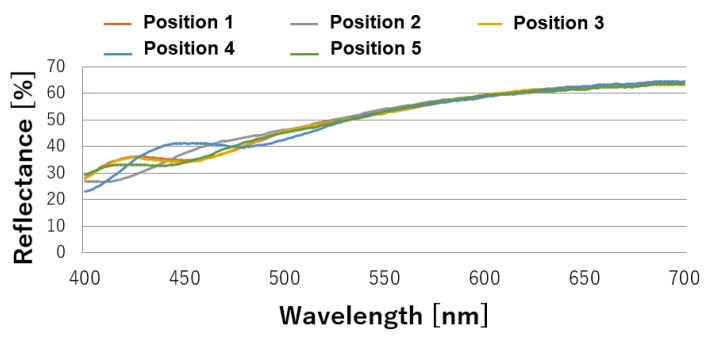
Reflectance in the visible light region for the Al roll mold.

**Figure 4:**
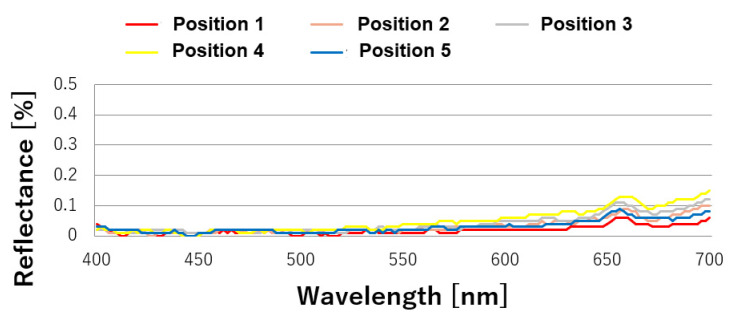
Reflectance of the proposed GC roll mold in the visible light region.

**Figure 5:**
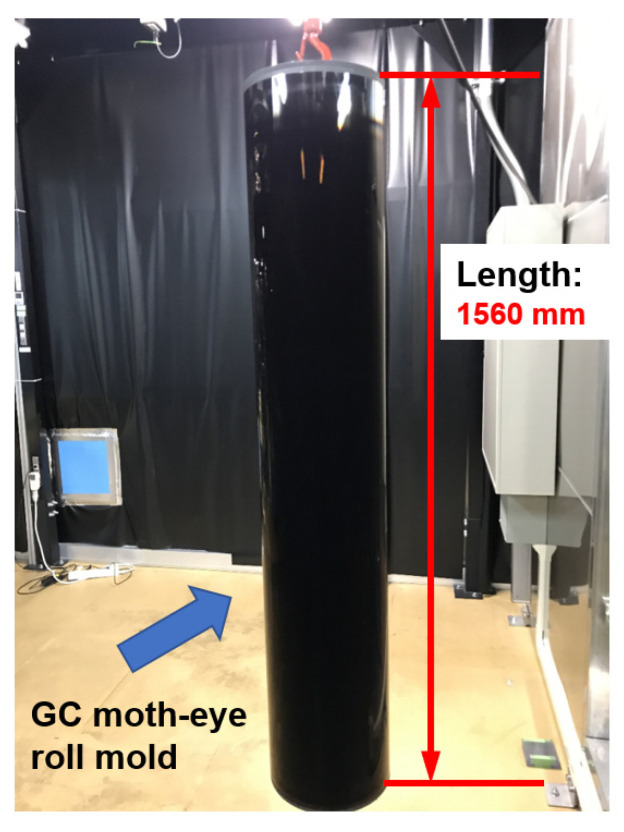
Appearance of the GC roll mold. A photograph of the proposed GC roll mold, which shows a low black reflection over the entire roll surface.

The reflectance results at five points at intervals of 340 mm inside 100 mm at both ends of the aluminum roll mold are shown in Figure 3. Its reflectance did not decrease because the processed surface had a conical–concave shape and did not have a moth-eye structure. The transfer pattern formed using the mold had a moth-eye structure, and therefore, its reflectance decreased. There were also variations in the low-wavelength region (400–500 nm). The reflectance results at five points at intervals of 340 mm inside 100 mm at both ends of the GC roll mold are shown in Figure 4. A low reflectance of less than 0.05% was obtained at all five points in the visible light region, and the processing was uniform. A photograph of the proposed GC roll mold is shown in Figure 5. Black reflection was low over the entire surface of the roll, and the moth-eye structure could be uniformly formed over a large area. The processing conditions for the GC roll plate were identical to those described in Section 2.2. However, a processing time of 55 min was required to obtain a reflectance of 0.05%.

### 3.2. Moth-Eye-Structured Film Shape and Optical Properties

**Figure 6:**
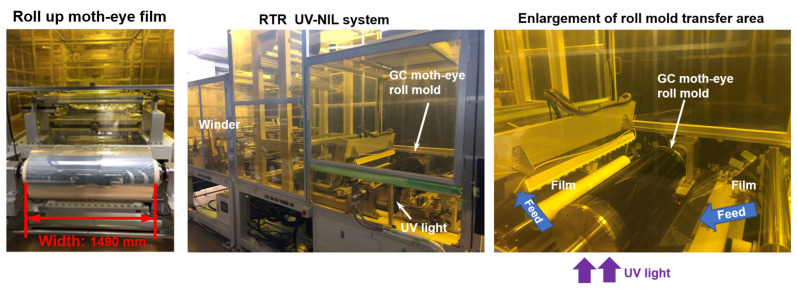
Photograph of the transferred 1490 mm wide proposed moth-eye-structured film (**left**), RTR UV-NIL system (**middle**), and enlargement of roll mold transfer area (**right**).

A photograph of a 1490 mm wide moth-eye-structured film transferred by RTR UV-NIL using a GC roll mold is shown in Figure 6. An explanation of the transfer motion of RTR UV-NIL is described in Section 2.3. When the roll diameter was 300 mm, the outer circumference of the roll was 942 mm. Using the RTR UV-NIL machine, a seamless and continuous transfer of the moth-eye-structured film was performed.

**Figure 7:**
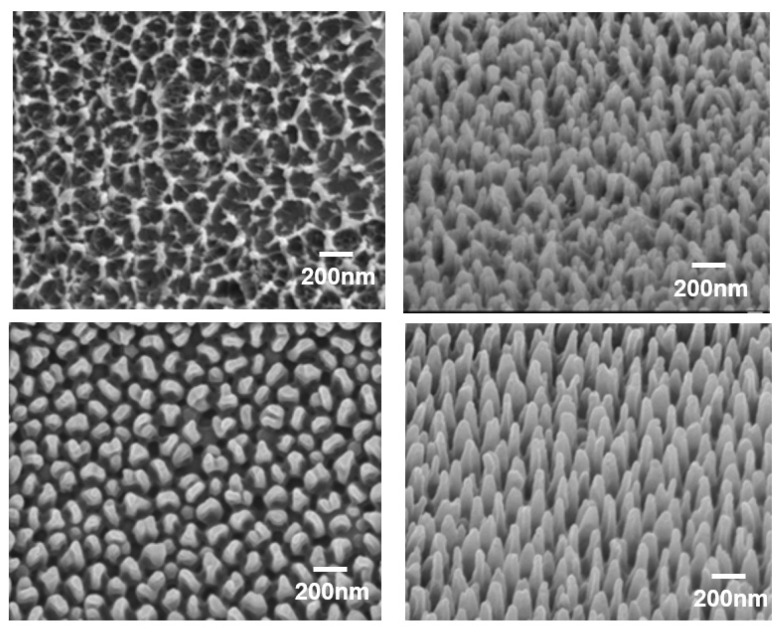
SEM images of the proposed moth-eye-structured films. Images of the film transferred from the GC roll mold: top view (**upper left**) and 45° tilted angle view (**upper right**). Images of the film transferred from the Al roll mold: top view (**lower left**) and 45° tilted angle view (**lower right**).

In the case of the Al roll mold, a moth-eye-structured film was prepared by RTR UV-NIL, and its shape was compared with that of the moth-eye-structured film prepared using the GC roll mold. The results are shown in Figure 7.

As shown in Figure 7, the diameter, pitch (distance between the centers of the needles), and height of the needle-shaped moth-eye structure transferred from the GC roll mold were measured at 10 points and averaged. The diameter was 65.5 ± 4.1 nm, and the pitch depth was 126.8 ± 4.8 nm. This diameter was larger than that of the GC roll mold, although the pitch was almost the same as that of the GC roll mold. The height was 337.5 nm ± 6.8, which was smaller than that of the GC roll mold. In the case of the Al roll mold, the diameter was 100 ± 2.8 nm, the pitch was 151.6 ± 2.8 nm, and the height was 425.6 ± 10 nm. These values were almost the same as those for the Al roll mold. Furthermore, as shown in Figure 7, when transferred from the GC roll mold, the resin entered the gaps in the needle-like structure of the mold such that the needles did not become independent needles, and there were places where the needles were connected to each other. In addition, unlike the transfer shape from the Al roll mold, it can be seen that the needle-shaped shape had a random structure with many variations in diameter and pitch.

**Figure 8:**
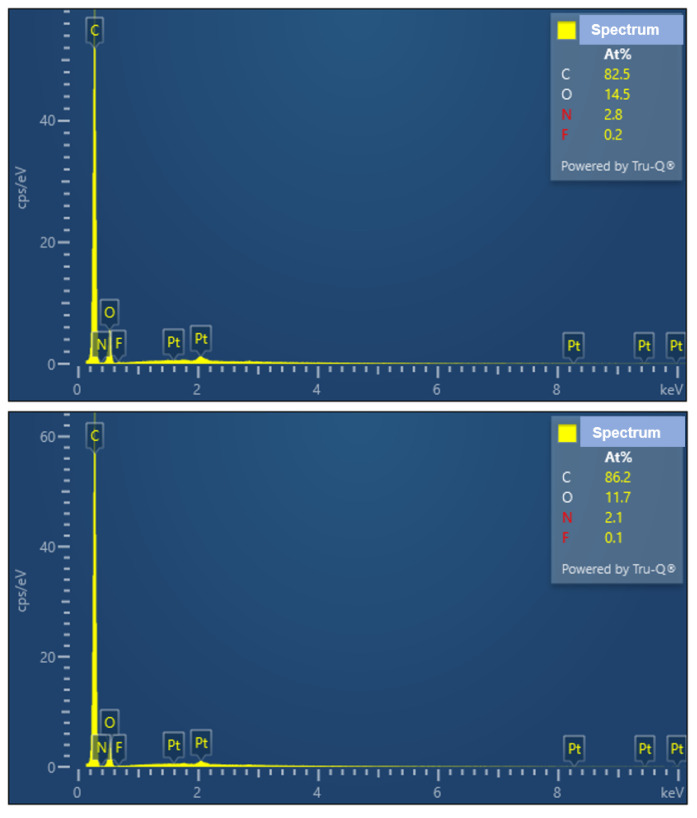
The energy dispersive X-ray spectroscopy (EDS) of moth-eye-structure films transferred from GC roll mold (**upper graph**) and transferred from Al roll mold (**lower graph**).

The EDS spectra in Figure 8 show the main carbon peak and a fluorine peak. The fluorine peak originated from the fluorine-based mold-release agent that was mixed with the resin. The fluorine ratios calculated from the EDS spectra were 0.2% from the GC roll mold and 0.1% from the Al roll mold.

**Figure 9:**
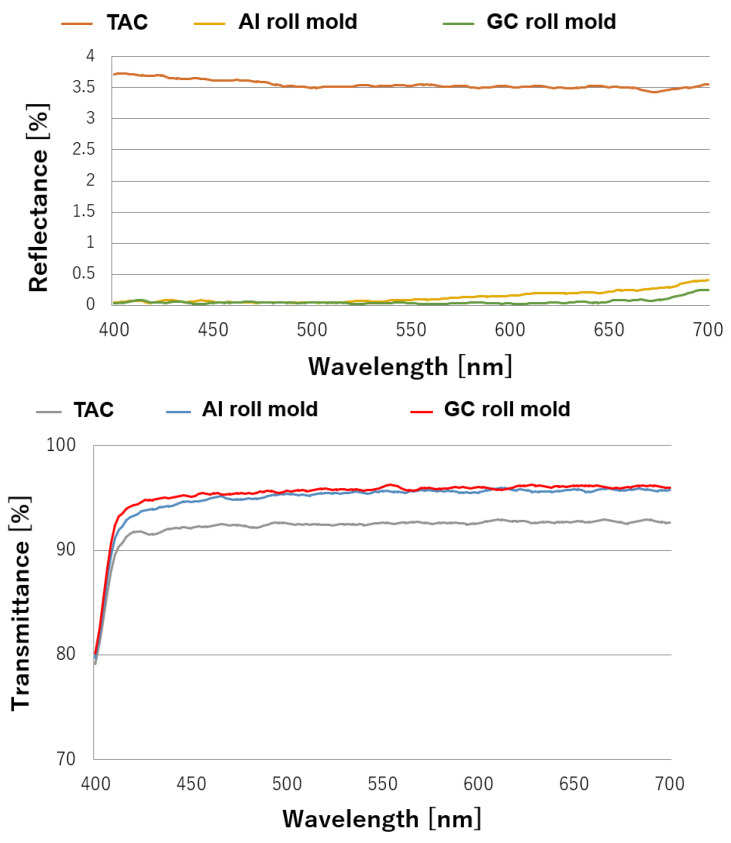
Reflectance (**upper graph**) and transmittance (**lower graph**) characteristics of the moth-eye-structured film from the GC roll mold, the moth-eye-structured film from the Al roll mold, and the TAC film.

The reflectance and transmittance characteristics of the TAC (the base film of the moth-eye-structured film) and moth-eye-structured films transferred from the Al and GC roll molds in the visible light wavelength region are shown in Figure 9.

As shown in Figure 9, the reflectance of the moth-eye-structured film was less than 0.1%, and its transmittance was 93%. In addition, the moth-eye structure obtained using the GC roll mold had a slightly lower reflectance and higher transmittance than that fabricated using the Al roll mold, and its performance was better. Despite the small differences in the reflectance and transmission between the films, we hypothesized that the moth-eye structure obtained using the Al roll mold was larger than that obtained using the GC roll mold in terms of both diameter and pitch. In addition, the moth-eye structure obtained using the Al roll mold was ordered, whereas that obtained using the GC roll mold had random positions. We believe that a smaller pitch, smaller diameter, and position randomness can promote low reflectivity.

### 3.3. Reflectance, Contact Angle, Haze, and Producibility Comparison of Moth-Eye-Structured Films

Further comparisons of the two types of moth-eye-structured films are summarized in Table 1. Table 1 shows the average value and standard deviation (SD) of five moth-eye films prepared using the two methods. The film fabricated using the GC roll mold (hereafter denoted as the GC moth-eye film) had a smaller reflectance than the moth-eye-structured film fabricated using the Al roll mold (hereafter denoted as the Al moth-eye film) at wavelengths of 450 and 550. However, at a wavelength of 700 nm, the Al moth-eye film had a smaller reflectance than the GC moth-eye film.

In the Yxy color system, Y represents brightness, and x (from 0 to 1) and y (from 0 to 1) represent color; these two values are represented as chromaticity coordinates. A diagram with the chromaticity coordinates x and y as the coordinate axes is referred to as an xy-chromaticity diagram. In the xy-chromaticity diagram, the color becomes more vivid as it moves outside. This method of describing colors can express not only object colors but all colors and is used to precisely describe colors. The Y value of the GC moth-eye film was lower than that of the Al moth-eye film. This implies that the GC moth eye exhibited a low brightness and suppressed reflectance compared to the Al moth-eye film. The x and y values of the GC and Al moth-eye films were almost identical, indicating achromaticity.

In the L* a* b* color system, the L* value represents brightness; the higher the L* value, the brighter the color. Color is represented by a* b*, and when both a* and b* are zero, a sample is achromatic. A more positive a * value indicates more redness; a more negative a* indicates more greenness; a more positive b* indicates more yellowness; and a more negative b* indicates more blueness. The L* value of the GC moth-eye film was lower than that of the Al moth-eye film. This indicates that the GC moth-eye film presented a lower brightness and suppression of reflectance. The a* and b* values of the GC and Al moth-eye films were close to zero, indicating that both films were achromatic. Overall, the reflectance properties of the GC moth-eye film were superior to those of the Al moth-eye film.

**Figure 10:**
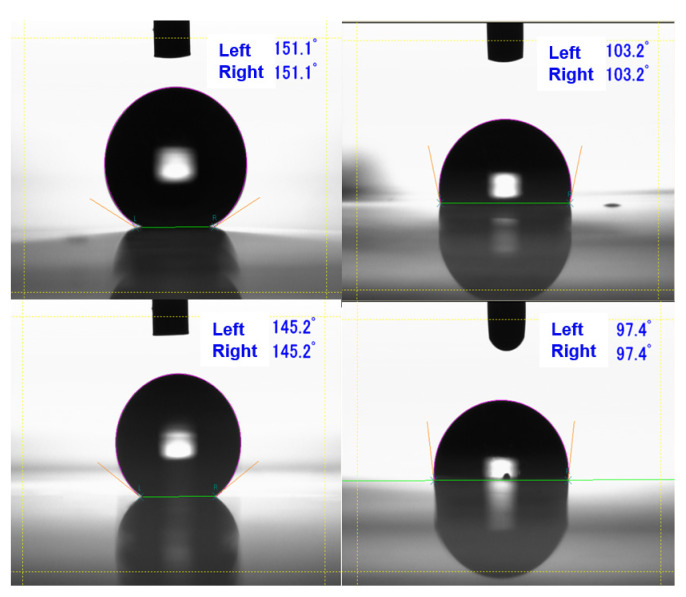
The CA photos of GC moth-eye film (**upper**) and Al moth-eye film (**lower**). Left-side figures are water CAs and right-side figures are n-hexadecane CAs.

Figure 10 shows images of the contact angles (CAs) of water and n-hexadecane. Because the CAs on the right and left sides were almost the same, five CA values from the right side were chosen and are summarized in Table 1. The GC moth-eye film had a larger contact angle with water and n-hexadecane than the Al moth-eye film. This indicated that the GC moth-eye film was superior in terms of water and oil repellency. In particular, the GC moth-eye film exhibited superhydrophobicity (CA > 150°). The haze value of the GC moth-eye films was smaller than that of Al moth-eye films, indicating that the GC moth-eye films possessed superior properties. The small SD values in Table 1 indicate that both methods have excellent reproducibility.

The proposed GC moth-eye film was superior to the Al moth-eye film in terms of reflectance, hydrophobicity, and oil-repellent properties. We believe that the excellent reflectance and contact angle characteristics of the GC moth-eye film were because of its shape. As shown in Figure 7 and the standard variation (SD) values, the SD of the GC moth-eye film pitch was ±4.8 nm, and the GC moth-eye film had a random pitch and diameter and a smaller diameter than the Al moth-eye film. This was effective in suppressing the reflection and high contact angle.

The films were then compared in terms of producibility. A GC roll mold may be produced using a vacuum process in a single chamber (Figure 1), enabling clean and efficient production. After the proposed GC thin film was formed, the moth-eye structure was processed in 55 min. Therefore, the total manufacturing time, including evacuation, was approximately 5 h.

However, the fabrication of an Al roll mold is a complex process. The process of anodization and wet etching was repeated five times to obtain the moth-eye structure, after which the last process was a final anodization. The Al roll was subjected to 11 bath changes, anodization, and pore-enlargement steps. Anodization required 120 s, and the pore-diameter-enlarging treatment required 900 s, both of which are short time periods. However, it took approximately 30 min to start the treatment after the temperature and concentration were kept constant in a bath. Assuming that one process takes 1 h, 11 h would be required to fabricate an Al roll. Furthermore, it is necessary to perform 11 wet processes, which are prone to errors and result in poor yield. Considering the simple process, production time, and yield of the GC roll mold, this was easier to produce than the Al roll mold.

### 3.4. Example of GC Moth-Eye Film Use

**Figure 11:**
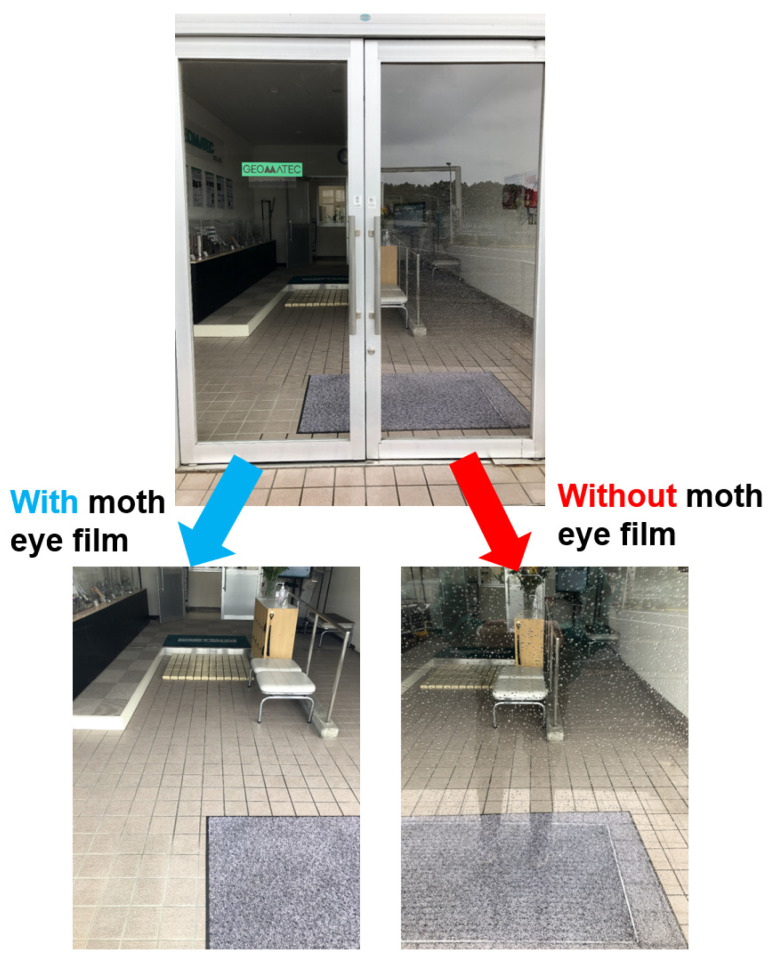
Visibility comparison with and without the proposed GC moth-eye-structured film.

An example of the actual use of the proposed large-area GC moth-eye film fabricated using RTR UV-NIL is shown in Figure 11. The left side of the photo shows glass with a moth-eye-structured film, and the right side shows glass without a moth-eye-structured film. There was no reflection on the glass with the film attached. Because the photograph was taken on a rainy day, water droplets adhered to the glass without the film. However, no water droplets adhered to the glass with the moth-eye-structured film attached, and the view through the window was not blocked.

As shown in Figure 11, a film with antireflection properties and excellent water repellency was produced in this study. This film may be used not only on the surface of flat panel displays but also in various other applications such as show windows, face shields, and ship windows.

## 4. Conclusions

To mass-produce a moth-eye-structured film with anti-reflection properties, a large-area roll mold was fabricated via two methods, namely an aluminum wet process and a GC dry process. The moth-eye-structured film was transferred by RTR UV-NIL using the two types of roll molds. Moth-eye-structured films were produced with good reproducibility using both methods. The Al moth-eye film had a regular structure, whereas the GC moth-eye film had random positions and was smaller in pitch and diameter than the Al moth-eye film. Because of these characteristics, the GC moth-eye film exhibited better reflection properties, haze, water repellency, and oil repellency than the Al moth-eye film.

There is high demand for the cost reduction and stable production of moth-eye films that may be used for various purposes, and it is essential to increase the size of the roll molds used in their fabrication. It is difficult to stabilize the quality and reduce the cost of conventional porous alumina molds because of the complexity of the required process. Conversely, GC thin films may be produced using any base material, and all processes can be completed in dry conditions using a vacuum film-forming apparatus.

In addition, once the film is set in a vacuum film-forming apparatus, processing up to the formation of the moth-eye plate is completed in the vacuum, which stabilizes the film quality.

In the future, the range of use for moth-eye films is expected to further expand, and the establishment of an approach for the stable manufacturing of large roll molds using GC thin films will significantly contribute to the anti-reflection-film market.

## Figures and Tables

**Table 1:** Comparison of reflectance, contact angle, and haze of moth-eye-structured films fabricated using the GC roll mold and Al roll mold.

		Reflectance Properties									Contact Angle [°]		Haze
		450 nm	550 nm	700 nm	Y	x	y	L*	a*	b*	Water	n-Hexadecane	[%]
		[%]	[%]	[%]									
GC moth-eye film	Average	0.085	0.16	0.26	0.17	0.37	0.35	−2.31	1.13	2.79	150	103	0.32
	SD	0.02	0.033	0.026	0.032	0.012	0.009	0.89	0.25	0.57	0.66	0.53	0.05
Al moth-eye film	Average	0.17	0.22	0.24	0.22	0.31	0.32	−0.87	−0.28	0.049	145	97	0.352
	SD	0.07	0.033	0.025	0.029	0.03	0.038	0.64	0.79	2.63	0.29	0.91	0.016
